# Quantifying the effects of attacks on health facilities on health service use in Northwest Syria: a case time series study from 2017 to 2019

**DOI:** 10.1136/bmjgh-2024-015034

**Published:** 2024-09-28

**Authors:** Ryan Burbach, Hannah Tappis, Aula Abbara, Ahmad Albaik, Naser Almhawish, Leonard S Rubenstein, Mohamed Hamze, Antonio Gasparrini, Diana Rayes, Rohini J Haar

**Affiliations:** 1 London School of Hygiene & Tropical Medicine, London, UK; 2 Johns Hopkins Bloomberg School of Public Health, Baltimore, Maryland, USA; 3 Infectious Disease, Imperial College Healthcare NHS Trust, London, UK; 4 Syrian American Medical Society, Washington, District of Columbia, USA; 5 Assistance Coordination Unit, Gaziantep, Turkey; 6 Syrian Public Health Network, London, UK; 7 Center for Public Health and Human Rights, Johns Hopkins Bloomberg School of Public Health, Baltimore, Maryland, USA; 8 Public Health Environments and Society, London School of Hygiene and Tropical Medicine, London, UK; 9 School of Public Health, Division of Epidemiology, University of California Berkeley, Berkeley, California, USA

**Keywords:** Public Health, Epidemiology

## Abstract

**ABSTRACT:**

**Background:**

Throughout the Syrian conflict, the Syrian government has intentionally attacked health facilities, violating International Humanitarian Law. Previous studies have qualitatively described health system disruptions following attacks on healthcare or established associations between armed conflict and health service utilisation, but there are no quantitative studies exploring the effects of health facility attacks. Our unprecedented study addresses this gap by quantifying the effects of health facility attacks on health service use during the Syrian conflict.

**Methods:**

This retrospective observational study uses 18 537 reports capturing 2 826 627 consultations from 18 health facilities in northwest Syria and 69 attacks on these facilities. The novel study applies case time series design with a generalised non-linear model and stratification by facility type, attack mechanism and corroboration status.

**Results:**

The study found significant, negative associations between health facility attacks and outpatient, trauma and facility births. On average, a health facility attack was associated with 51% and 38% reductions in outpatient, RR 0.49 (95% CI 0.43 to 0.57) and trauma consultations, RR 0.62 (95% CI 0.53 to 0.72), the day after an attack, with significant reductions continuing for 37 and 20 days, respectively. Health facility attacks were associated with an average 23% reduction in facility births, the second day after an attack, RR 0.77 (95% CI 0.66 to 0.89), with significant reductions continuing for 42 days.

**Conclusions:**

Attacks on health facilities in northwest Syria are strongly associated with significant reductions in outpatient, trauma and facility births. These attacks exacerbate the adverse effects of armed conflict and impede the fundamental right to health. The findings provide evidence that attacks on health facilities, violations of international humanitarian law by themselves, also negatively affect human rights by limiting access to health services, underscoring the need to strengthen health system resilience in conflict settings, expand systematic reporting of attacks on healthcare and hold perpetrators accountable.

**WHAT IS ALREADY KNOWN ON THIS TOPIC**

- Existing literature described significant relationships between armed conflict and negative health system outcomes (both in Syria and globally); documented increases in reported attacks on healthcare over time; summarised interviews describing attacks on healthcare from those who experienced them and estimated the scope of attacks on healthcare in Syria. However, there is a dearth of quantitative research on the effects of attacks on healthcare. We reviewed literature that explored the relationship between attacks on healthcare and health systems, especially studies that provided a quantitative estimate of the effects of attacks on healthcare and health outcomes or health system outputs. We searched Medline and Google Scholar for peer-reviewed manuscripts published between 1 January 2000 and 31 May 2023 using the search terms: (“attacks on health*” OR “violence against health*” OR “health facility attack*”) AND (“health*” OR “humanitarian*”) AND (“conflict” OR “war”). The search produced studies providing qualitative analysis of healthcare worker experiences with attacks on health, the effects of general conflict on the health system and descriptive summaries comparing attacks on healthcare in different conflicts but did not yield any results quantifying the relationship between attacks on healthcare and the health system.

**WHAT THIS STUDY ADDS**

- To our knowledge, this is the first study to quantify the effects of attacks on healthcare on health service use, separate the effects of attacks on healthcare from the effects of general conflict and apply the case time series design in a humanitarian context. By applying this design and controlling for seasonality, variation in consultations by day of the week, holiday periods and general conflict intensity, we demonstrated that health facility attacks are significantly associated with sustained reductions in health service use in the days, weeks and months following an attack.

**HOW THIS STUDY MIGHT AFFECT RESEARCH, PRACTICE OR POLICY**

- The findings from this study have implications for both policy and practice. From a policy perspective, this study provides evidence that health facility attacks result in sizeable and sustained disruptions to health service use and may impede the fundamental right to health. This information can be used to further perpetrator accountability and improve legal protections for civilians. From a practice perspective, this study provides healthcare providers and humanitarian response agencies with an estimate of the magnitude and duration of health service disruption following an attack on health services to inform future response activities and improve health system resilience.

## Background

 Attacks on healthcare during armed conflict are violations of International Humanitarian Law (IHL), but there is a dearth of quantitative research on the effects of these attacks. Quantifying disruptions to health service delivery could provide evidence of violations of the human right to health and inform future humanitarian response.^1^ Globally, attacks on healthcare are increasingly reported with 2022 accounting for the most attacks in the past 10 years.^2^ Previous research has identified negative associations between armed conflict and health outcomes, detailed healthcare worker experiences following attacks on health, and described attacks on health in different conflicts.^3^,^6^ However, existing research has neither quantified the short-term to intermediate-term effects of attacks on healthcare on the health system, nor separated the effects of general armed conflict from the effects of attacks on healthcare. Providing quantitative evidence on the effects of attacks on health will help clarify whether these attacks not only breach the law but also impede the human right to health by disrupting health service use and could strengthen legal protections, advance perpetrator accountability and contribute to health system resilience.

In March 2011, the Syrian government violently responded to peaceful uprisings against the Syrian government, sparking a protracted armed conflict and devastating adverse health system effects. The conflict has killed more than 350 000 civilians, forcibly displaced more than half the preconflict population of 22 million people and caused over $120 USD billion in infrastructural damage.^7^,^9^ Reduced vaccination coverage has resulted in measles and polio outbreaks, diseases that had previously been eliminated from Syria for decades.^10^ Qualitative studies have reported health workforce attrition due to the conflict, patient fear in seeking health services and experiences of service disruptions and delays across the health sector.^11^,^13^ Numerous, well-documented war crimes have occurred during the conflict, including intentional and strategic attacks on health facilities, healthcare workers, ambulances and other health infrastructure.^14 15^ Physicians for Human Rights (PHR) have documented 601 attacks on healthcare in Syria since 2011 and the killing of more than 900 healthcare workers.^16^

Compounding this violence, the Syrian Ministry of Health withdrew health service provision in northwest Syria (an area where around 4 million people shelter) early in the conflict resulting in local organisations and humanitarian responders developing an ad hoc health network to provide health services over the past decade. This led to a fragmented network of organisations coordinated through the Turkey-based, WHO-led Health Cluster and includes both local and international humanitarian organisations.^17^ Since 2014, northwest Syria has been reliant on cross-border aid subjected to UN Security Council resolutions, meaning the aid that reaches the population in this area is heavily restricted. The Syrian American Medical Society (SAMS), a non-governmental organisation (NGO), providing medical aid in conflict-affected areas of Syria, is one of the many organisations operating within this fragmented network to respond to the health needs of the population in this area. During the study period, SAMS managed 38 (18%) of the 206 health facilities in Aleppo, Hama and Idlib governorates as per the Global Health Cluster Health Resources and Services Availability Monitoring System. Many of the attacks on healthcare in northwest Syria have targeted health facilities in this network. This study quantifies and characterises the short-term and intermediate-term impacts of attacks on healthcare on health service use during the Syrian conflict.

## Methods

### Study scope

This study explores the relationship between attacks on healthcare and health consultations at SAMS health facilities in Aleppo, Hama and Idlib governorates in northwest Syria between 1 March 2017 and 31 December 2019. The timeframe reflects the period during which SAMS documented daily outpatient and trauma consultations at 38 health facilities in three governorates and facility births at 13 health facilities in Aleppo and Idlib governorates.

### Patient and public involvement

As this study uses retrospective, aggregate health facility consultation data, the public and patients were not involved in this study. Given the conflict context, frequent migration and privacy concerns, no attempt was made to follow up with individual patients or the public.

### Data sources

#### Exposure variables: health facility attack data

In Syria, NGOs began documenting attacks on healthcare in 2011. The study uses datasets compiled by PHR and SAMS, respectively. PHR developed a comprehensive registry of attacks on healthcare and a systematic process to corroborate these attacks.^16^ After scouring official reports, media coverage and social media for reports of attacks on healthcare, PHR systematically assessed the credibility of the source and triangulated multiple credible sources before declaring an attack corroborated. PHR documented 89 corroborated attacks on healthcare in Aleppo, Hama and Idlib governorates during the analysis period from 1 March 2017 through 31 December 2019. The SAMS dataset, based on local reporting, captured 284 attacks on healthcare during the same period in the same governorates.

The PHR and SAMS datasets were cross-referenced to explore consistency between the two sources. Facility names and locations were manually matched with the SAMS health facility register to add a unique facility identifier, confirm appropriate geographic locations and link the health facility attacks with daily consultation datasets. While both datasets broadly defined attacks on healthcare, this study includes only direct attacks on a SAMS-managed physical health facility, henceforth health facility attacks. Attacks reported on targets other than a physical health facility or those without a date, inconsistent geographic locations, or that occurred when the facility had already been closed for more than 1 day prior to the attack were omitted. On manual review, the SAMS dataset was more likely to list several strikes on the same day on the same facility as individual attacks, whereas the PHR dataset was more likely to document one attack, combining the smaller strikes into one event. To address differences in how a start or end to an attack may be defined between the datasets, a binomial exposure variable was created to indicate whether a health facility attack occurred at each facility each day regardless of the number of individual attacks reported. The results include stratification by corroborated and uncorroborated attacks, which reflected facility-days PHR reported an attack as well as facility-days which SAMS reported an attack, but PHR did not, respectively. After applying these criteria and limiting entries to the study period and locations for which outcome data were available, the final analysis dataset included 69 health facility attacks on 18 health facilities in 14 communities in Aleppo, Hama and Idlib governorates. The analysis dataset included the attack date, attack mechanism, health facility type, geographic administrative classifications, corroboration status and a unique identifier for each health facility. The attack mechanism was available for 28 (41%) of the health facility attacks in the final dataset and was grouped into three categories: aerial bombardments, explosions from ground weapons and small arms attacks.

#### Outcome variables: health consultation data

SAMS health facilities provided data on outpatient consultations, trauma consultations and facility births during the study period. SAMS developed a Microsoft SQL-based reporting system to track daily consultations at their facilities beginning in March 2017. This system captured the facility name, administrative levels, outpatient consultations, trauma consultations and facility births at each facility, documenting 98 550 daily consultation reports from 88 health units during the analysis period in Aleppo, Hama and Idlib governorates. Facility births were defined as live and stillbirths delivered in the health facility, including both normal and Caesarean section deliveries. We combined health units that fell within the same health facility, as outpatient and inpatient units often reported separately, and removed facilities that did not routinely report study outcome variables. These criteria were applied to produce a consultation dataset containing 34 286 daily reports and 4 479 035 outpatient consultations, trauma consultations and facility births across 16 hospitals and 22 primary healthcare centres (PHC) in Aleppo, Hama and Idlib governorates. These facilities were assigned unique identifiers to link with exposure and covariate datasets. The linked dataset was subset to include only those 18 health facilities that experienced at least one health facility attack during the study period and had daily consultation data available to produce the final analysis dataset.

#### General conflict event data

We used publicly available data cataloguing conflict events in Syria from the xSub: Cross-National Data of Sub-National Violence.^18^ xSub compiles registries tracking armed conflict events sourced from media reports and other secondary data sources, such as the Armed Conflict Location and Event Data project and the Uppsala Conflict Data Program. The xSub dataset includes the event date, spatial coordinates, event type and location confidence estimate. Events categorised as protests, riots, strategic movements or similar non-violent or non-military events were dropped from the dataset.

A nearest neighbour analysis with community point locations as indicated in UNOCHA geodatabases was used to assign each armed conflict event to a community with appropriate administrative levels and codes that facilitate linkages to the exposure and outcome datasets (geodatabases included as an annex).^19 20^ In total, the analysis dataset included 36 900 conflict events in 1497 communities in Aleppo, Hama and Idlib governorates. Conflict data were aggregated by day and community in both a simple count of conflict events and a conflict intensity score we developed. This score was created to account for presumed differences in effect severity by type of weaponry used. For example, the effects of an airstrike were expected to be more severe than effects from small-arms fire. The weighting was based on previous studies estimating destruction and mortality resulting from various weaponry.^21 22^ Airstrikes, suicide bombings and shelling were assigned a weight of 4; mortars, rocket propelled grenades, and similar weaponry were assigned a weight of 2 and small-arms fire was assigned a weight of 1. These weights were then summed for the number of conflict events and weaponry used in each community each day. Large-scale attacks are more likely to be captured and reported than small-scale attacks, thus this study may underestimate conflict intensity in areas with few aerial bombardments, but heavy ground battles.

### Statistical analysis

Preliminary descriptive analysis explored trends in exposure and outcome variables by consultation type, day of the week and time elapsed since an attack to inform the study statistical methods. We estimated the association between health facility attacks and each outcome variable by applying a case time series design to model facility-specific series through a quasi-Poisson regression to account for overdispersion observed in the data. The case time series design was developed for environmental epidemiology but is well suited to the time-variant exposures and facility-based outcomes in this study. This design and model address time-variant exposures and confounders, offer a self-matched design for routine health facility consultation data and account for the anticipated lagged effects following a health facility attack.^22^ The exposure-outcome associations were modelled through distributed lag non-linear models with lags of 1–90 days. This lag was selected based on exploratory analysis. The lag was extended to 120 days for the stratified analysis on corroboration status to capture the longer duration of observed effects. The models included terms to control for seasonal trends, daily conflict intensity, day of the week and Ramadan periods. A spline with 4 degrees of freedom per year was used to control for seasonal consultation trends. Subsequent models for outpatient consultations were stratified by attack corroboration status and attack mechanism. Stratified models were not developed for other outcome variables due to insufficient sample sizes once stratified.

## Results

### Descriptive analysis

**Figure 1:**
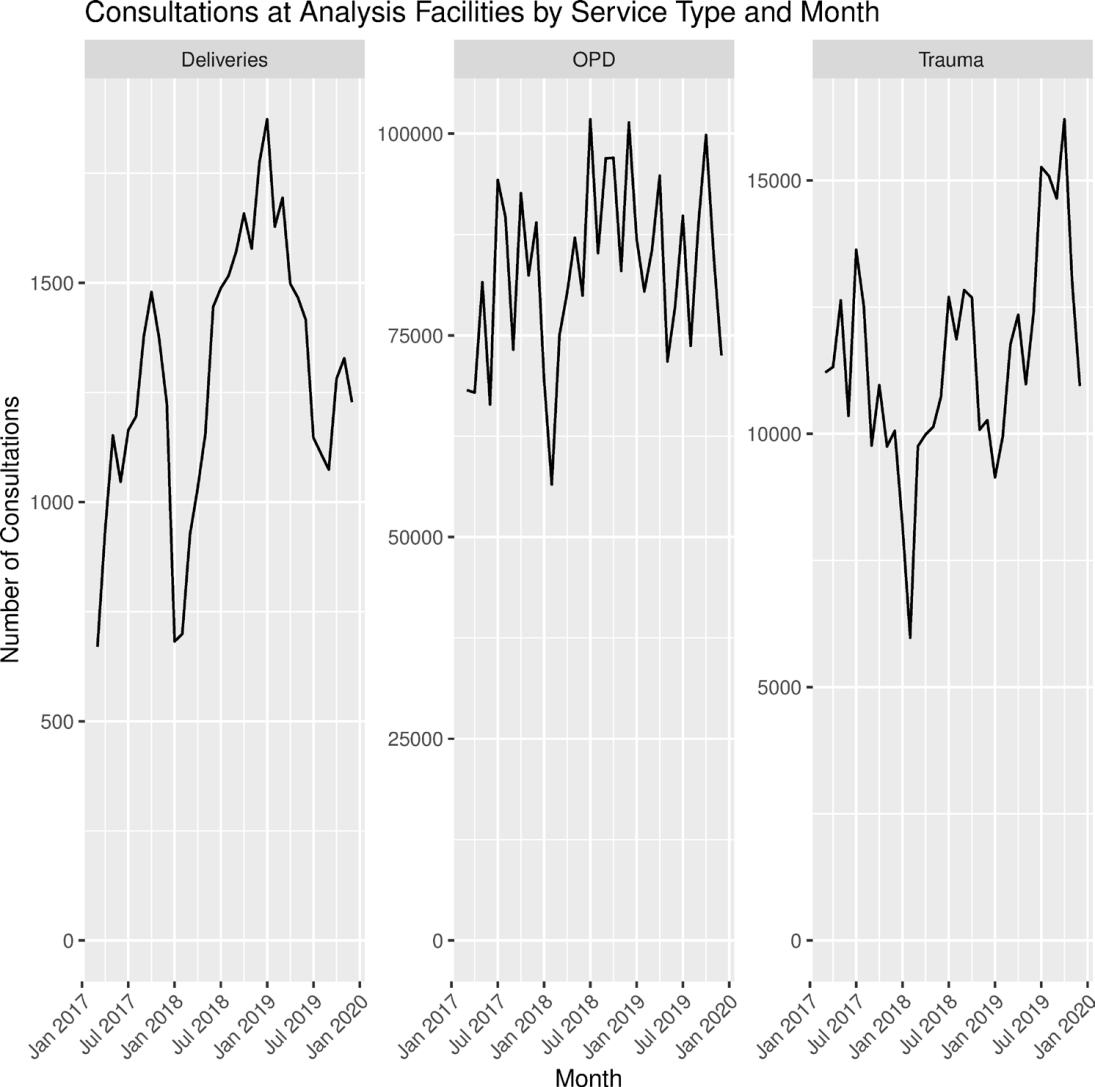
Consultations at analysis facilities by service type and month for maternal deliveries, outpatient consultations (OPD) and trauma consultations.

**Figure 2:**
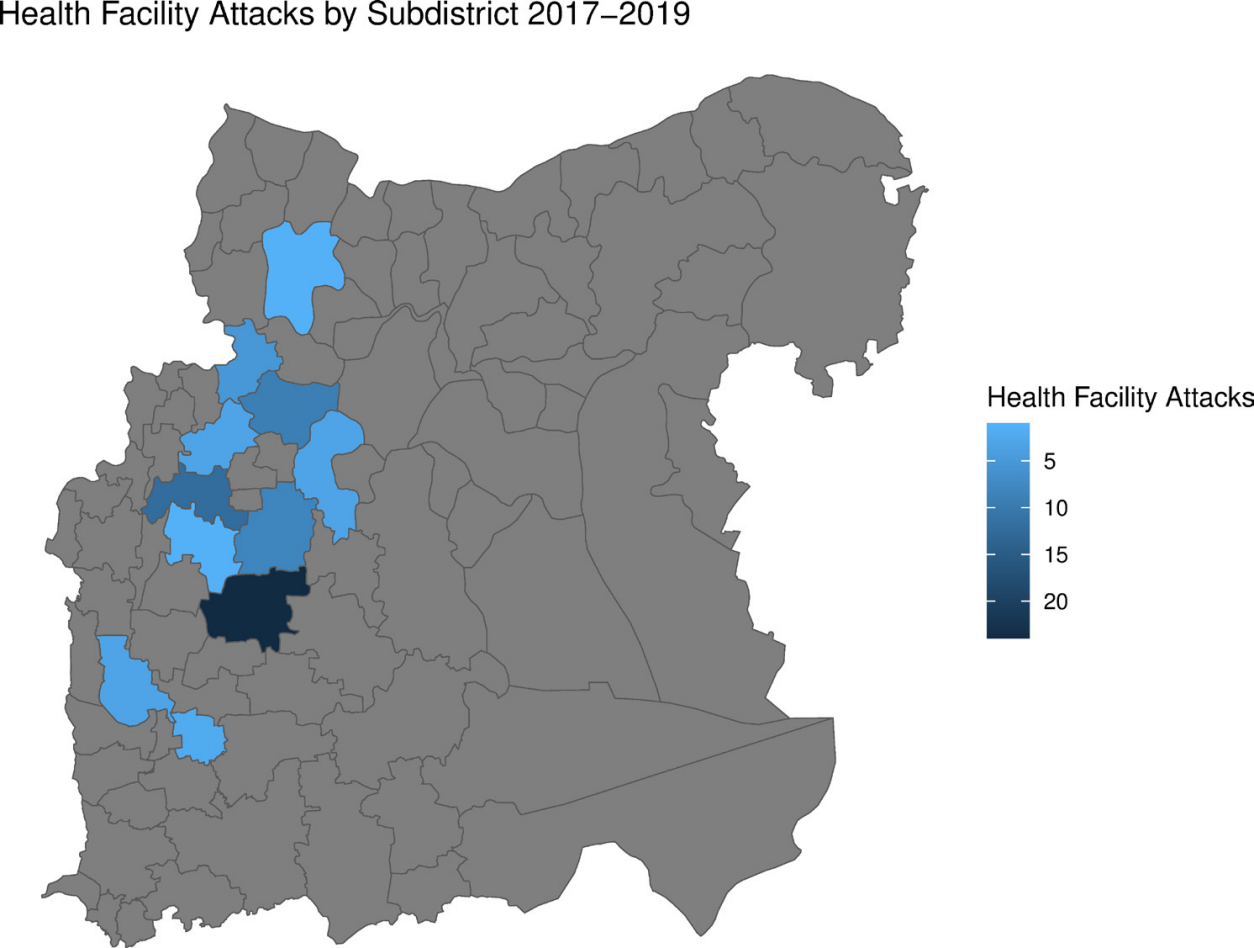
Health facility attacks by subdistrict 2017–2019.

**Figure 3:**
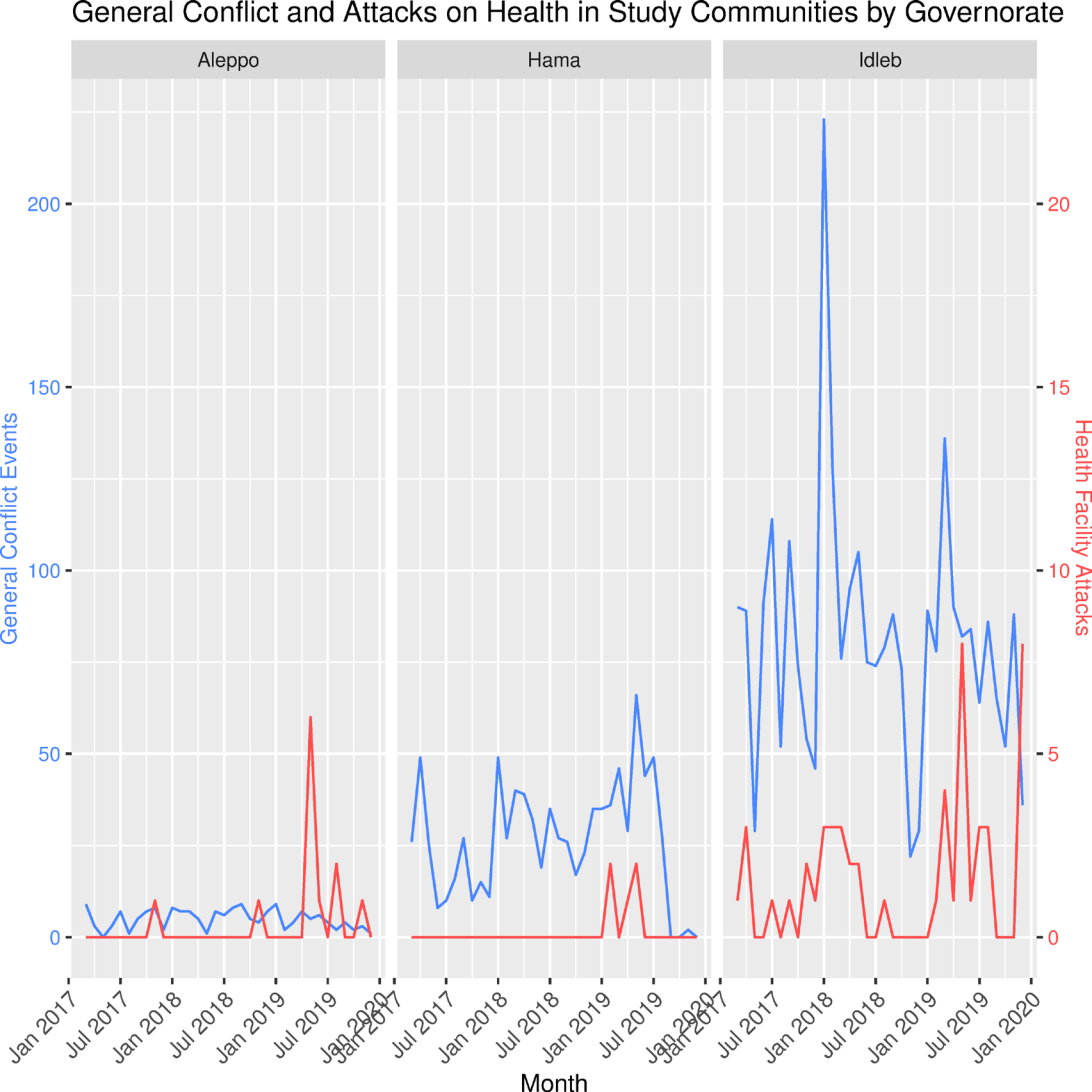
General conflict and attacks on health in study communities by governorate.

The analysis dataset contained 18 537 daily reports of 2 826 627 outpatient consultations from 18 health facilities in three governorates and 69 health facility attacks; 15 531 reports of 389 134 trauma consultations from 15 health facilities in three governorates and 58 health facility attacks; and 6216 reports of 43 885 facility births from six health facilities in two governorates and 33 health facility attacks. Hama was not included in the facility births dataset as no health facilities in Hama both reported facility births and experienced a health facility attack in the study period. In total, Idlib, Aleppo and Hama accounted for 75%, 23% and 2% of all consultations in the final dataset, respectively. Figure 1 depicts the consultation trends by type during the study period. Figure 2 shows the geographic distribution of health facility attacks by subdistrict. Figure 3 presents health facility attack and general conflict event trends over time by governorate. Individuals involved in the medical response indicated the decrease in all consultation types in early 2018 shown in figure 1 may be linked to the sharp increase in general conflict events in Idlib during the same period shown in figure 3.^23^ Ten health facility attacks in the analysis were corroborated by PHR and 59 were uncorroborated. The attack mechanism was available for 28 attacks: 17 aerial attacks (60.7%), 7 explosions (25%) and 4 small arms attacks (14.3%). In these 14 communities during the same period, there were 3832 general conflict events.

### Case time series design with general non-linear models

**Figure 4:**
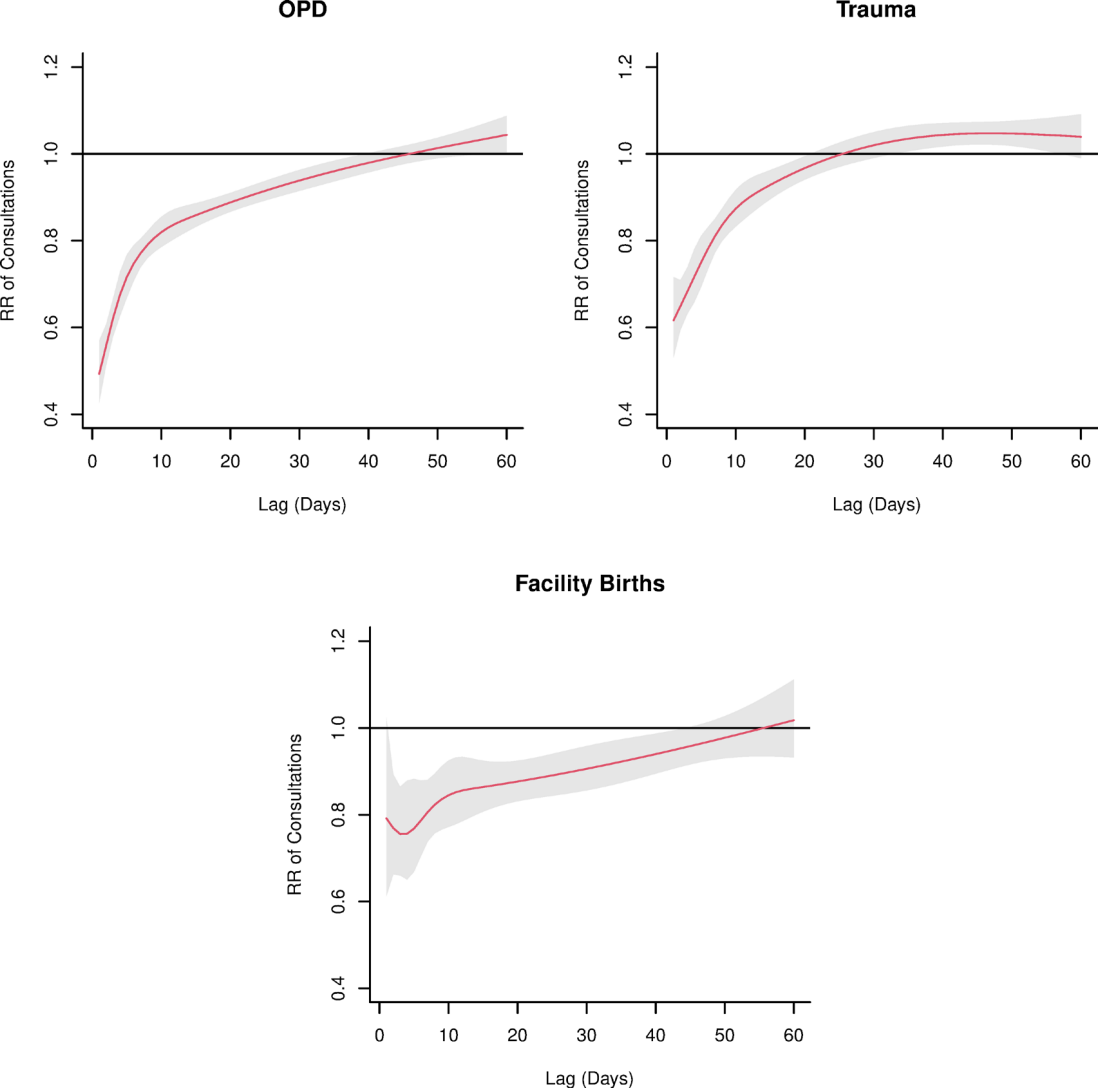
Relative risk of utilisation change by time and type of consultation, i.e. outpatient consultations (OPD) and trauma consultations and facility births.

We observed strong negative associations between health facility attacks and health consultations with significant reductions in outpatient, trauma and facility birth consultations for extended periods after an attack. In the day following a health facility attack, facilities experienced on average a 51% reduction in outpatient consultations and 38% reduction in trauma consultations: RR 0.49 (95% CI 0.43 to 0.57) and RR 0.62 (95% CI 0.53 to 0.72), respectively. Significant negative associations between health facility attacks and consultations were observed for 37 days after an attack for outpatient consultations and 20 days after an attack for trauma consultations with diminishing magnitude over time (figure 4). No significant association was observed the day after an attack for facility births, but significant negative associations were observed from the second day after an attack, RR 0.77 (95% CI 0.66 to 0.89), and every subsequent day until 42 days after an attack.

**Figure 5:**
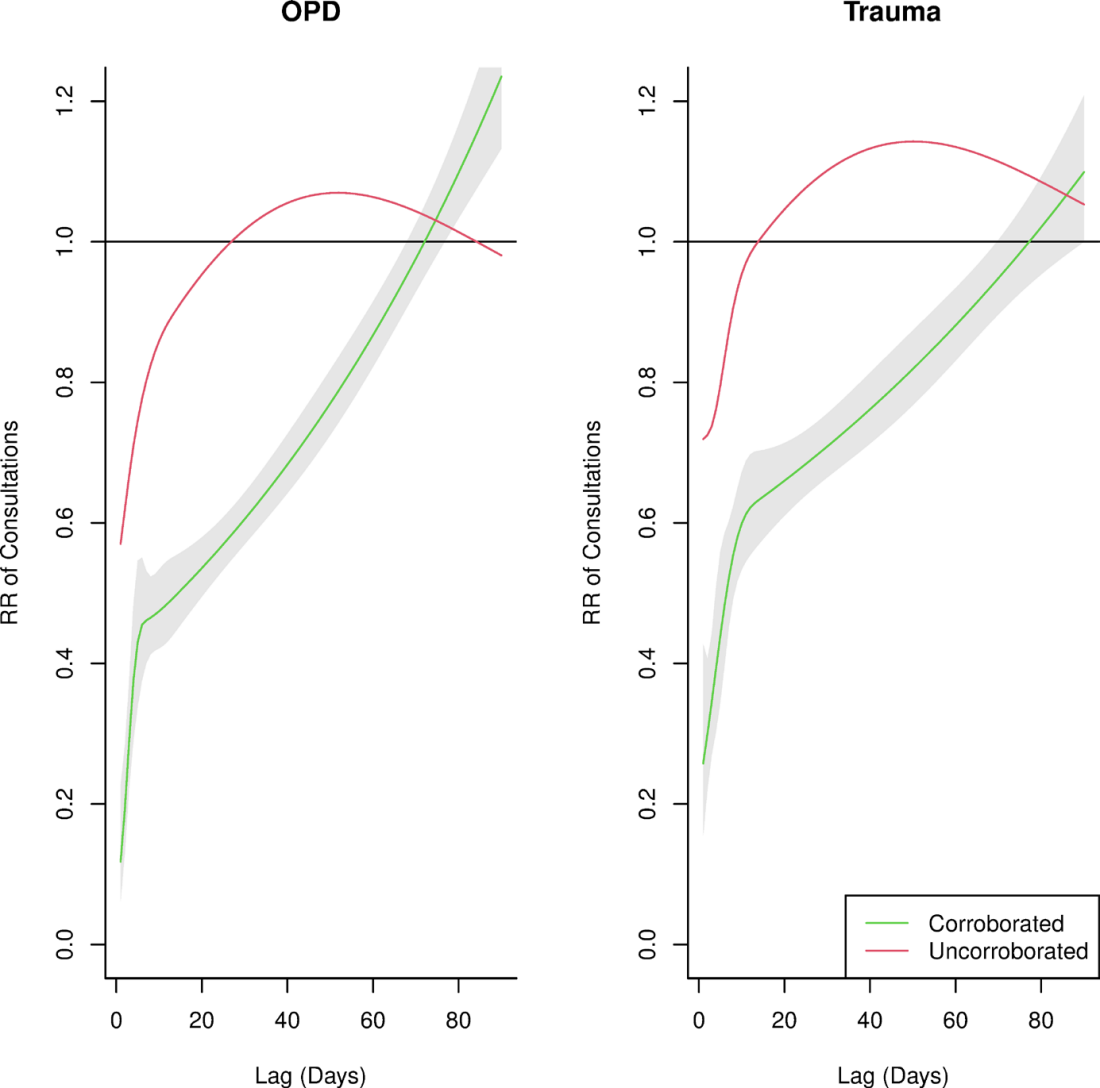
Relative risk of utilisation change by corroboration status by outpatient consultations (OPD) and trauma consultations.

Health facility attacks corroborated by PHR had a greater effect than those that were not corroborated on outpatient; RR 0.12 (95% CI 0.06 to 0.23) versus RR 0.57 (95% CI 0.49 to 0.66), respectively; and trauma consultations; RR 0.26 (95% CI 0.16 to 0.43) versus RR 0.72 (95% CI 0.61 to 0.84), respectively; in the day following an attack (figure 5). Corroborated health facility attacks were associated with significant reductions in outpatient and trauma consultations for 67 and 68 days, respectively, while uncorroborated attacks were associated with significant reductions for 22 and 9 days, respectively.

Aerial bombardments and small arms attacks were associated with significant reductions in outpatient consultations; RR 0.30 (95% CI 0.21 to 0.43) and RR 0.42 (95% CI 0.25 to 0.72), respectively, 1 day after a health facility attack. Significant reductions in outpatient consultations were observed for 116 days after an aerial bombardment and for 5 days after a small arms attack. No significant association was observed between explosions from land-based weapons and outpatient consultations in the 8 days following an attack, but they were positively associated with outpatient consultations from 9 days, RR 1.11 (95% CI 1.01 to 1.23) to 107 days after an attack. Attacks on both PHCs and hospitals were associated with significant reductions in outpatient consultations; RR 0.25 (95% CI 0.15 to 0.42) and RR 0·54 (95% CI 0.46 to 0.64) the day after an attack and continuing for 50 and 30 days, respectively. Models for trauma consultations and facility births were not produced due to insufficient sample sizes.

## Discussion

This study provides clear quantitative evidence that attacks on health facilities in Syria reduce access to and utilization of health services even when controlling for armed conflict in the surrounding community. We found that attacks are strongly associated with significant reductions in outpatient consultations, trauma consultations and facility births in the days and weeks after an attack. A health facility attack was associated with an average 51% reduction in outpatient consultations the day following a health facility attack and were significantly reduced for 37 days after an attack. Trauma consultations dropped an average of 38% the day following an attack and remained significantly reduced for 20 days after an attack. There was no significant reduction in facility births the day after an attack but dropped an average of 23% the second day after an attack and remained significantly reduced through day 42 after an attack. These sizeable and lasting disruptions to health service use impede the essential right to health and introduce additional barriers to populations already dealing with increased morbidity and mortality risk from armed conflict and associated factors.

This study addresses a major research gap for attacks on healthcare and, to our knowledge, is the first study to quantitatively estimate the effects of health facility attacks on health system outputs. The quantitative results of our study support qualitative studies describing the devastating legacy of attacks on healthcare on clinical staff and health service delivery during the Syrian war.^11 12^ Other studies have provided descriptive summaries of attacks on healthcare, estimated the negative association between armed conflict and health service utilisation in Syria, or increased morbidity and mortality risk from armed conflict more globally but had not quantitatively explored relationships between attacks on healthcare and the health system.^3 24 25^ Our study provides causal links between attacks on healthcare and negative health outcomes and separates the direct effects of health facility attacks from the effects of armed conflict, both key gaps identified in the published literature. This research is a critical step in understanding the impact of these violations of IHL and estimates disruptions to health service access and delivery that impede the right to health.^26^ As attacks on healthcare proliferate, this study provides a methodological platform for further exploration of related humanitarian and health system effects in other contexts.^2^

Our findings offer a more nuanced understanding of attacks on healthcare, demonstrating that neither attacks, nor their impacts are homogeneous. Attacks corroborated by PHR and attacks using aerial bombardment were associated with stronger and long-lasting effects than for uncorroborated attacks and other attack mechanisms, respectively. Corroborated attacks may be associated with stronger effects given the likelihood that large-scale, explosive attacks are documented and reported by multiple sources than smaller, less visible attack mechanisms, such as small arms fire. We acknowledge that attacks with small arms are less likely to be captured and reported in both the corroborated and uncorroborated datasets and future research should explore corroboration processes and the underlying reasons for the sizeable difference in effect by corroboration status, whether due to the likelihood that large-scale attacks are documented, and thus more readily corroborated, or other currently unexplained factors. The stronger, long-lasting effects for aerial bombardments match assumptions that this attack mechanism may cause more substantial damage.^15 27^

Despite these findings, our study may in fact underestimate the effects of aerial bombardments given the drastic, protective measures that were taken to fortify health facilities in northwest Syria,^28^ many of which were fortified before prior to the study period. Prior to 2017, organisations, including SAMS, developed strategies to strengthen health system resilience, such as fortifying health facilities or building them in caves and improving supply chain mechanisms. The geographic proximity of northwest Syria to functioning supply chains near the Turkish border, allowing for faster restocking and reestablishing health services, may also introduce bias when compared with other parts of Syria. Future attacks on health research could explore whether our findings on effects by weaponry are consistent in contexts where the health system may be unprepared or ill-equipped to take these protective measures or in conflicts with fewer aerial bombardments.

Health facility attacks were not associated with a significant reduction in facility births the day immediately after an attack but were negatively associated with beginning the second day after an attack. Recent qualitative studies and conversations with our local research team suggested that this may be due to limited options for maternity care and shortages of skilled health personnel, leading women to continue with their delivery at the hospital where they had planned to give birth, even if it had been attacked the day prior. In the days and weeks after an attack, many women may have had time to rearrange their labour plan and would be able to schedule services at alternative locations.^29 30^ Future research could explore whether this hypothesis is accurate or whether there are changes in home delivery frequency after a facility is attacked.

As this was a retrospective, observational study, the analyses were subjected to data quality challenges. SAMS operated and was able to provide data on 18% of the 206 health facilities operating in northwest Syria and only 9% of the 206 health facilities were included in the analysis due to exposure, outcome and covariate data availability. Having access to only one organisation’s consultation data could introduce bias as patients might have sought care at nearby health facilities in other health networks. This study is unable to measure consultation trends throughout the health system, thus preventing any attribution of consultations lost to health facility attacks. Furthermore, mortality data were unavailable to explore whether there were any immediate or lagged mortality effects associated with consultation reductions. This study was unable to explore the main factors following a health facility attack that led to reduced consultations. For example, reduced staffing levels, destroyed medical stocks, debilitated hospital rooms and infrastructure or community inability to access the attacked facility are all possible explanatory factors for reduced consultations following health facility attacks.

This analysis would not have been possible without the daily consultation data diligently collected by SAMS. Preliminary exploratory analyses using less robust methods showed that most of the effects of health facility attacks would have been masked had monthly consultation data been used. Daily data allowed for investigation of short-term effects of health facility attacks, which was particularly important as facilities generally recovered to preattack consultation counts within weeks after an attack. While difficult, daily or weekly reporting of consultation data in locations experiencing attacks on healthcare is highly recommended to provide sufficient data to study attack effects on health service use. We recommend that formal technical guidance and systematic data collection systems for attacks on healthcare with linkages to routine humanitarian datasets containing population, armed conflict and other covariate data be developed to facilitate documentation of health facility attack effects.

As suggested in other literature, there is a need and opportunity for systematic data collection in conflict areas experiencing attacks on health,^6 26^ which requires appropriate global guidance and standards. Coordination around attacks on health research and health service utilisation data earlier in the conflict could have made available data from a larger proportion of the health service providers operating in northwest Syria, addressing a key limitation of this study. Similar research could be undertaken more easily in the future with clear guidance on key exposure, outcome and confounding variables to collect, the frequency with which to collect them and linkages between common datasets in humanitarian settings, such as population, displacement, health facility mapping, disease burden and health service utilisation data. Methodological guidance would also be beneficial, similar to previous documents produced on how to estimate population denominators or mortality in humanitarian contexts.^31 32^ For example, we conducted a novel application of the case time series model in the humanitarian context, which could have further application in research on attacks on health, given its self-matched design and overall ability to manage time-variant exposures and covariates.

The absence of population denominators to calculate health service utilisation rates is a major study limitation. Population estimates were reviewed but were not incorporated into the models due to changing administrative boundaries during the analysis period. Local actors leading population estimation activities indicated the number of administrative areas used to estimate population increased from approximately 6000 in 2016 to approximately 8000 in 2018. While population-based consultation rates would have offered a stronger measure to estimate change in service use than crude counts, the analytical design incorporating daily lags offers the benefit of presenting both the immediate effects in the days following a health facility attack, when changes in population and other potential time-variant confounders would likely be minimal, and the long-term effects that may be subjected to additional scrutiny.

Our models cannot account for several unobserved variables to which changes in consultation counts could be attributed. Health service use could have been affected by changes in health-seeking behaviour, spatial access, transportation infrastructure, household purchasing power, health seeking within other health networks or facility consultative capacity due to staff reductions. Staff attrition in northwest Syria has been documented throughout the war^30 33^ and documenting staffing levels in the aftermath of an attack would help with understanding of the underlying reasons for daily consultation decline following an attack. Furthermore, funding cuts were reported to have taken place at some SAMS facilities, but the timing of these funding changes was not documented. These funding cuts could have led to changes in service delivery and corresponding utilization data that we could not account for in the models. Our models indicated high degrees of overdispersion, ranging from 4.01 to 44.7, despite assuming a quasi-Poisson distribution. This could indicate an unobserved predictor variable with a large effect on consultations. Future research should explore the effects of these aforementioned factors, and others, and whether they address the overdispersion in the current models.

## Conclusion

This study describes the devastating effect health facility attacks have on health consultations, even when adjusting for armed conflict in the surrounding community. The results suggest that attacks on health facilities, direct violations of IHL, also impede the fundamental right to health. Our findings, the first to quantify the relationship between attacks on healthcare and health service use, underscore the need to hold perpetrators accountable, provide information that humanitarian response organisations can use for practical response operations and serve as a template for future data collection and research practices in areas experiencing attacks on health.

While this study falls short of providing a direct causal link between attacks on health and civilian mortality, it provides evidence farther along this causal pathway than has previously been available and provides a strong evidence base from which to build. We encourage future attacks on health research to include: (1) attributable effects of health facility attacks on health outcomes or mortality, (2) population denominators given frequent population migration, (3) explanations for the absence of a measurable effect of health facility attacks on facility births the day following an attack, (4) effects of unmeasured covariates in similar models, (5) effects by weaponry used in different contexts, (6) better understanding of effect differences by corroboration or reported status and (6) protective effects of fortifying health facilities against aerial bombardments.

To facilitate additional research on the health impacts of attacks on health, we recommend that formal technical guidance and systematic data collection systems be developed to ensure comprehensive datasets are available. Linkages between routine humanitarian datasets should be established and well defined within the technical guidance. This information should be made widely available to humanitarian actors and shared immediately on the onset of armed conflict and before attacks on health occur, where possible, to encourage systematic, consistent data collection that can be used in research to hold future perpetrators accountable.

## Data Availability

Data are available in a public, open access repository.

## References

[1] International Committee of the Red Cross (2016). Geneva conventions of 1949 and additional protocols, and their commentaries. https://www.icrc.org/applic/ihl/ihl.nsf/vwTreaties1949.xsp.

[2] (2023). Ignoring red lines: violence against health care in conflict 2022. https://reliefweb.int/report/ukraine/ignoring-red-lines-violence-against-health-care-conflict-2022.

[3] Ekzayez A, Alhaj Ahmad Y, Alhaleb H (2021). The impact of armed conflict on utilisation of health services in north-west Syria: an observational study. Confl Health.

[4] Chukwuma A, Ekhator-Mobayode UE (2019). Armed conflict and maternal health care utilization: Evidence from the Boko Haram Insurgency in Nigeria. Soc Sci Med.

[5] Ramadan M, Tappis H, Uribe MV (2021). Access to primary healthcare Services in Conflict-Affected Fragile States: a subnational descriptive analysis of educational and wealth disparities in Cameroon, Democratic Republic of Congo, Mali, and Nigeria. Int J Equity Health.

[6] Briody C, Rubenstein L, Roberts L (2018). Review of attacks on health care facilities in six conflicts of the past three decades. Confl Health.

[7] OHCHR A/HRC/50/68: civilian deaths in the Syrian Arab Republic - report of the United Nations high commissioner for human rights. https://www.ohchr.org/en/documents/reports/ahrc5068-civilian-deaths-syrian-arab-republic-report-united-nations-high.

[8] Syrian Arab Republic, ReliefWeb (2021). Humanitarian needs overview: Syrian Arab Republic (March 2021) [EN/AR]. https://reliefweb.int/report/syrian-arab-republic/2021-humanitarian-needs-overview-syrian-arab-republic-march-2021-enar.

[9] AP NEWS (2018). The latest: UN says civil war has cost Syria $388b in damage. https://apnews.com/article/aa0aaa2c44cd430196f572227b45c150.

[10] Mehtar S, AlMhawish N, Shobak K (2021). Measles in conflict-affected northern Syria: results from an ongoing outbreak surveillance program. Confl Health.

[11] al-Nahhas H, Fricke A, Rayes D (2023). She pays the highest price: the toll of conflict on sexual and reproductive health in Northwest Syria. https://phr.org/our-work/resources/sexual-and-reproductive-health-in-northwest-syria/.

[12] al-Nahhas H, Fricke A, Moran A (2021). The survivors, the dead, and the disappeared: detention of health care workers in Syria, 2011-2012. https://phr.org/our-work/resources/the-survivors-the-dead-and-the-disappeared/.

[13] DeJong J, Ghattas H, Bashour H (2017). Reproductive, maternal, neonatal and child health in conflict: a case study on Syria using Countdown indicators. BMJ Glob Health.

[14] Fouad FM, Sparrow A, Tarakji A (2017). Health workers and the weaponisation of health care in Syria: a preliminary inquiry for The Lancet –American University of Beirut Commission on Syria. The Lancet.

[15] Visual Investigations Team (2020). Russia tapes: healthcare and civilians under attack in Syria, 2019. https://www.nytimes.com/spotlight/visual-investigations-russia-syria.

[16] Physicians for Human Rights A map of attacks on health care in Syria. https://phr.org/our-work/resources/a-map-of-attacks-on-health-care-in-syria/.

[17] Al-Abdulla O, Ekzayez A, Kallström A (2023). Health system recovery in Northwest Syria–challenges and operationalization. *Humanit Soc Sci Commun*.

[18] Center for Political Studies xSub: cross-national data of sub-national violence. https://cps.isr.umich.edu/project/xsub-cross-national-data-of-sub-national-violence/.

[19] OCHA Syria Syrian arab republic: populated places. Syrian Arab Republic.

[20] Syrian Arab Republic Subnational administrative boundaries. https://data.humdata.org/dataset/cod-ab-syr.

[21] Coupland RM, Samnegaard HO (1999). Effect of type and transfer of conventional weapons on civilian injuries: retrospective analysis of prospective data from Red Cross hospitals. BMJ.

[22] Guha-Sapir D, Rodriguez-Llanes JM, Hicks MH (2015). Civilian deaths from weapons used in the Syrian conflict. BMJ.

[23] Yacoubian M (2018). Idlib: the last major battle in the Syrian civil war. https://www.usip.org/blog/2018/09/idlib-last-major-battle-syrian-civil-war.

[24] Haar RJ, Risko CB, Singh S (2018). Determining the scope of attacks on health in four governorates of Syria in 2016: Results of a field surveillance program. PLoS Med.

[25] Wagner Z, Heft-Neal S, Bhutta ZA (2018). Armed conflict and child mortality in Africa: a geospatial analysis. The Lancet.

[26] Afzal MH, Jafar AJN (2019). A scoping review of the wider and long-term impacts of attacks on healthcare in conflict zones. Med Confl Surviv.

[27] Winter C (2018). Russian strikes in Syria kill 18,000. https://www.dw.com/en/russian-airstrikes-in-syria-reportedly-killed-18000-people/a-45702091.

[28] Elamein M, Bower H, Valderrama C (2017). Attacks against health care in Syria, 2015–16: results from a real-time reporting tool. *The Lancet*.

[29] Bashour H, Kharouf M, DeJong J (2021). Childbirth Experiences and Delivery Care During Times of War: Testimonies of Syrian Women and Doctors. *Front Glob Womens Health*.

[30] Alhaffar M, Hamid A, Douedari Y (2022). “We are trying to live in a normal way, but nothing is normal about us anymore…”: a qualitative study of women’s lived experiences of healthcare in opposition-controlled areas of Syria. BMJ Glob Health.

[31] Abdelmagid N, Checchi F (2018). Estimation of population denominators for the humanitarian health sector guidance for humanitarian coordination mechanisms. https://healthcluster.who.int/docs/librariesprovider16/meeting-reports/lshtm-population-guidance-ghc-nov2018.pdf?sfvrsn=3a425173_1&download=true.

[32] Roberts L, Checchi F Interpreting and using mortality data in humanitarian emergencies. https://odihpn.org/publication/interpreting-and-using-mortality-data-in-humanitarian-emergencies/.

[33] Heisler M, Baker E, McKay D (2015). Attacks on Health Care in Syria--Normalizing Violations of Medical Neutrality?. N Engl J Med.

